# 2940. Weaving Federal and Private Funding to Achieve Hepatitis C Elimination Goals at Syringe Services Programs in Rural Kentucky

**DOI:** 10.1093/ofid/ofad500.179

**Published:** 2023-11-27

**Authors:** Nicholas Van Sickels, Jaime Soria, Alice C Thornton, Amanda B Wilburn, Jana Collins

**Affiliations:** University of Kentucky College of Medicine, Lexington, Kentucky; University of Kentucky, Lexington, Kentucky; The University of Kentucky, Lexington, Kentucky; The University of Kentucky, Lexington, Kentucky; University of Kentucky, Lexington, Kentucky

## Abstract

**Background:**

Addressing the syndemic of HIV, Hepatitis C (HCV), and Opioid Use Disorder in rural Kentucky requires unique approaches to funding, assessment, and treatment. The Kentucky Income Reinvestment Project (KIRP) leverages Ryan White, CDC, and private funding to provide harm reduction services, including HIV and Hepatitis C testing, across the majority of Kentucky's Syringe Services Programs (SSPs). We sought to compare HCV evaluations between SSPs with KIRP support versus SSPs without KIRP support. Additionally, we describe early results from a pilot study providing direct treatment at the SSP from 2 KIRP-supported counties (Figure 1).

**Figure:**
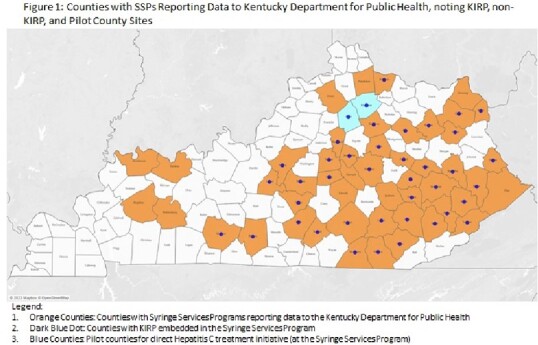

**Methods:**

We performed a cross-sectional review of data reported to Kentucky Department for Public Health from March 2021 to March 2023. OraQuick® HCV rapid antibody tests were used for screening. We compared the number of eligible clients seen and tested for HCV, the seropositivity, and referral rates for treatment. Additionally, we report pilot outcomes from two counties, where patients were treated at the SSP. Study data was managed using the REDCap data capture tool. Chi-squared tests were used to describe statistical differences between groups, with analyses performed using Stata 17 (College Station, TX: Stata Corp LLC).

**Results:**

Over a 24-month period, KIRP sites saw and tested a higher volume and percentage of clients than non-KIRP sites (18.3% versus 4.9%, p< 0.001; see Figure 2). HCV seropositivity was similar between KIRP and non-KIRP sites (31.5% versus 23.2%, p=0.058). HCV treatment referrals were greater at KIRP sites, with 646/654 (98.8%) referred compared to 26/29 (89.7%) referred at non-KIRP sites (p=0.009). Pilot counties received 107 referrals from May 2022 to March 2023. 51/107 were evaluated; nine had negative RNA testing, 17 did not show, and 25 completed one visit. To date, 5 patients achieved SVR, and 5 are undergoing treatment (Figure 3).

**Figure:**
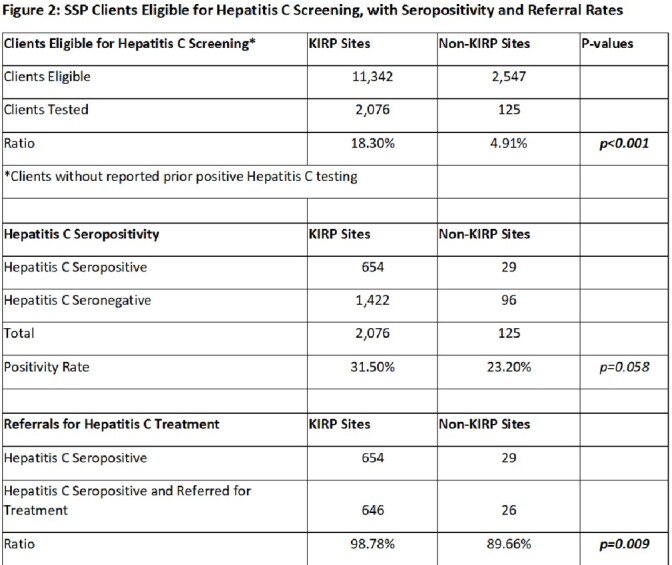

**Figure:**
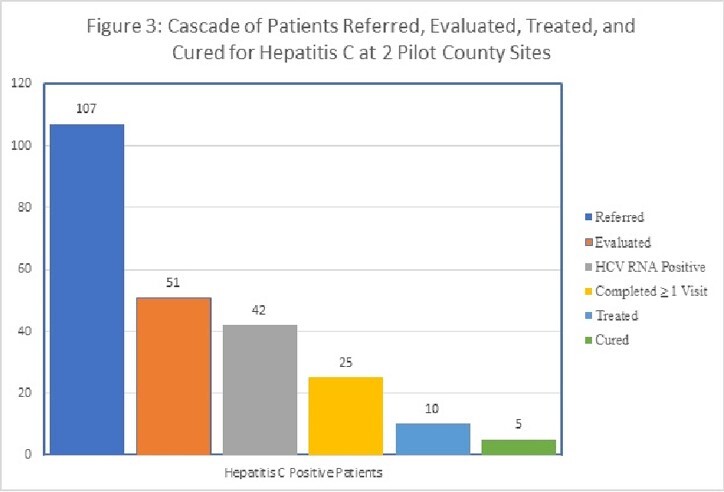

**Conclusion:**

Our study demonstrates significantly higher rates of HCV testing, linkage, and referral at KIRP-supported SSPs in Kentucky. Overlaying funding from the Ryan White Care Act with CDC and private entities allows for status neutral assessments for HIV and HCV. Partnerships for direct treatment of HCV at the SSP, especially in more rural areas, are crucial to the HCV elimination strategy.

**Disclosures:**

**All Authors**: No reported disclosures

